# Shared Decision Making in the Psychiatric Inpatient Setting: An Ethnographic Study about Interprofessional Psychiatric Consultations

**DOI:** 10.3390/ijerph19063644

**Published:** 2022-03-18

**Authors:** Caroline Gurtner, Christa Lohrmann, Jos M. G. A. Schols, Sabine Hahn

**Affiliations:** 1 Applied Research and Development in Nursing, Department of Health Professions, Bern University of Applied Sciences, 3008 Bern, Switzerland; sabine.hahn@bfh.ch; 2 Department of Health Services Research, Care and Public Health Research Institute (CAPHRI), Maastricht University, 6200 MD Maastricht, The Netherlands; jos.schols@maastrichtuniversity.nl; 3 Institute of Nursing Science, Medical University Graz, 8010 Graz, Austria; christa.lohrmann@medunigraz.at; 4 Department of Family Medicine & Care and Public Health Research Institute (CAPHRI), Maastricht University, 6200 MD Maastricht, The Netherlands

**Keywords:** shared decision making, clinical practice, psychiatric inpatient setting, interprofessional psychiatric consultations, psychiatric disorder, mental illness, communication, patient involvement, patient-centered care, participant observation

## Abstract

Shared decision making is increasingly receiving attention in health care and might improve both the quality of care and patient outcomes. Nevertheless, due to its complexity, implementation of shared decision making in clinical practice seems challenging. This ethnographic study aimed to gain a better understanding of how psychiatric inpatients and the interprofessional care team interact during regular interprofessional psychiatric consultations. Data were collected through participant observation on two different psychiatric wards in a large psychiatric hospital in Switzerland. The observation focused on the contextual aspects of interprofessional patient consultations, the communication and interaction as well as the extent to which patients were involved in decision making. Participants included patients, psychiatrists, junior physicians, nurses, psychologists, social workers and therapists. We observed 71 interprofessional psychiatric consultations and they differed substantially in both wards in terms of context (place and form) and culture (way of interacting). On the contrary, results showed that the level of patient involvement in decision making was comparable and depended on individual factors, such as the health care professionals’ communication style as well as the patients’ personal initiative to be engaged. The main topics discussed with the patients related to pharmacotherapy and patient reported symptoms. Health care professionals in both wards used a rather unidirectional communication style. Therefore, in order to promote patient involvement in the psychiatric inpatient setting, rather than to focus on contextual factors, consultations should follow a specific agenda and promoting a bidirectional communication style for all parties involved is strongly recommended.

## 1. Introduction

In recent years, the concepts of patient- and people-centered care have gained in importance and have been promoted by various countries through legislation policies as well as by health organizations [1,2]. The concept of patient-centered care (PCC) focuses on the individual needs of patients and encourages their participation in health-related decisions, whereas person centeredness goes one step further and puts the person in the center and not the diagnosis or the symptoms. Both concepts may offer potential benefits to patients and health care professionals. On the one hand, patients gain profit from better health-related outcomes, enhanced health literacy and satisfaction with care [3], while on the other hand, health care professionals experience improved job satisfaction and the quality of care may increase while the overall costs decrease [4].

Shared decision making (SDM) is also a promising approach to promote patient and person centeredness in clinical practice. All three concepts share common elements: for instance, they emphasize the importance of the health care professional–patient relationship, the importance of a mutual exchange of information and patient involvement in care [4,5]. Especially in the context of the psychiatric inpatient setting, the concept of SDM has been identified to be useful to move from provider-centered to people-centered care by supporting inpatients to “achieve their own goals” and by focusing on their resources rather than on problems and deficits during the therapeutic process [6,7,8].

Although the positive factors of SDM and PCC on patient experience and outcomes have been widely discussed in the literature, implementation in clinical practice seems challenging and difficult, and it often fails due to the different preconditions of the diverse health care settings, including contextual and cultural aspects regarding the involvement of patients in care [5,8,9]. Additionally, the biomedical model of mental illness indicates considerable influence on clinical psychiatric practice. For instance, patients with acute or severe mental illnesses are not always recognized by professionals as having adequate decision-making capacity, and health care professionals—and especially physicians—tend to take over the authority of decision making for them [10,11,12]. This is clinically relevant, as over 40% of psychiatric inpatients are classified as “moderately to severely mentally ill” [13].

According to the WHO (2017), depression disorders and anxiety disorders are common, and depression is the major driver of suicidal deaths [14,15]. Depending on the severity of the symptoms, depression and anxiety disorders can lead to hospitalization and, along with schizophrenia and substance disorders, are among the most frequent psychiatric diagnoses leading to admission to a psychiatric hospital [15]. Today, the preferred approach to treatment in the clinical setting is based on pharmacotherapy together with complementary strategies, such as psychotherapy or stimulation therapies [16,17].

Considering the diversity of the patients’ individual situations, decision making in the acute psychiatric setting is often complex, also because the outcome of the decisions taken is usually not immediately apparent [18]. Despite promising developments in recent decades, current treatment approaches are only partially effective and mental illnesses, in addition to disease-related symptoms, also impose a significant personal and socio-economic burden on those affected [17]. This becomes apparent, for example, through the loss of residence or workplace, the loss of colleagues or friends or through social welfare dependency. Therefore, it is important to aim for a long-term perspective already in the acute phase of the disease during a hospital stay and involving patients in disease management [19]. In the context of SDM, therapeutic as well as personal and socio-economic decisions must be made, for example whether and when a patient can return to his or her residence and workplace, which follow-up treatments are recommended, or which disease-related behaviors should be addressed in psychotherapy [20,21,22]. Thus, regarding the efforts to promote SDM in psychiatric clinical practice, attention should be paid to situations involving social interactions and organizational processes, such as interprofessional psychiatric consultations, where the health care professionals and the patient communicate regarding the above-mentioned topics [12]. To date, interactions between the patient and the physician have been the focus of research, while the importance of other professions (e.g., nurses) in the context of SDM has received only little attention [10,23,24].

Interprofessional psychiatric consultations in Swiss hospitals, which usually take place once or twice a week, play a central role in everyday clinical practice and provide an important tool regarding information exchange, coordination, and provision of care as well as decision making [25,26]. In the context of decision making, patients could benefit from the interaction with various members from the interprofessional care team, for instance, to clarify treatment-related questions or to address their concerns and preferences along the spectrum of their disease management [23,27,28]. Therefore, the aim of this ethnographic study was to examine the nature of interactions and communication between health care professionals and patients during regular interprofessional psychiatric consultations. The study setting was chosen purposively, addressing contrasting and ward-related aspects (contextual and cultural factors) of interprofessional psychiatric consultations in a psychiatric hospital. The following research questions guided this study:

(1) How are psychiatric inpatients involved during interprofessional psychiatric consultations and how do they engage with the interprofessional care team regarding health-related decisions?
(2) What key topics do patients discuss with whom of the interprofessional care team?
(3) How do ward-related (contextual and cultural) aspects of interprofessional psychiatric consultations impact the communication between the patient and the interprofessional care team?

## 2. Materials and Methods

### 2.1. Design

A qualitative ethnographic design was chosen [29]. This study aimed to understand the social interactions and organizational process, i.e., the where and how of interprofessional patient consultations in the acute psychiatric inpatient setting. Data were collected by participant observation using a structured observation protocol and spontaneous conversations with the participants (patients and health care professionals) were documented through field notes. The Standards for Reporting Qualitative Research (SRQR) were used to guide the reporting of this study [30].

#### 2.1.1. Researcher Characteristics

The observer (first author) in the field had a scientific perspective as well as professional expertise. As a trained nurse with several years of professional experience in acute psychiatry, the observer already had a relationship with the field. This was also disclosed to the health care professionals being observed. However, the observer was situated in the background during the observation sequences and followed the events as a “distanced observer” [29]. This means that the observer (first author) did not interfere in the conversations with the patients and the health care professionals during the patient consultations. The observation itself was performed following a focused and systematic approach, addressing the research questions of this study.

#### 2.1.2. Context

This study took place in a large representative Swiss psychiatric hospital, which offers the full range of services for people with mental health problems, from outpatient, intermediate and acute inpatient services to rehabilitation. In the acute inpatient setting, the hospital’s management wants to promote a diversity of services offered, and therefore the individual ward managers can implement different treatment concepts. For this study, two acute psychiatric inpatient wards (A and B) were selected as a purposive sample for data collection [31]. Both showed a comparable group of patients being treated but a maximum variation regarding ward-related aspects, such as context and culture. The ward culture on the one hand is part of the context and is defined as “the way things are done in a particular setting” [32]. In this regard, the ward culture is not only influencing the health professional’s way of interacting and their decision-making style but can either be present as tacit knowledge or referring to a written ward concept [33]. On the other hand, the ward context refers to both, structural aspects of the interprofessional psychiatric consultations (e.g., the place in which the consultations took place) and person-related aspects of the health care professionals present (e.g., language, physical position and role) [27].

Information on the ward culture and on the context of interprofessional psychiatric consultations (Wards A and B) were obtained in advance of this study. For this purpose, the principal investigator contacted the two ward managers and conducted an informal interview with each of them. Both ward managers also provided a written ward concept which supplemented the data material of this study. In Ward A, a traditional medically oriented approach, based on the physicians’ leadership, was applied. This was pointed out in the ward concept as follows: “Patients are being treated according to the most recent scientific and clinical findings and frequent medical consultations are a central element of care in ward A”. Moreover, it was reported by the ward manager that interactions during the interprofessional patient consultations mainly take place between the physicians and the patient. Furthermore, the interprofessional psychiatric consultations in Ward A were traditionally held in the patient’s room.

In Ward B, the implemented ward concept is aimed at a strong focus on the importance of patients’ individual resources and coping with mental impairment. The interprofessional psychiatric consultations are therefore carried out in the form of a “treatment conference”, the aim of which is to offer opportunities for participative and goal-oriented cooperation between the interprofessional care team and the patient [34]. The interprofessional patient consultations in Ward B were held in a separate meeting room, where all participants sit in a circle.

#### 2.1.3. Recruitment

The health care teams on both selected wards were contacted in advance and invited to participate in an information session on behalf of this study. In addition, the nursing managers from Wards A and B supported the observer (first author) by distributing written study information to the targeted health professionals and obtained their informed consent of being observed on the allocated observation dates. All health professionals, who were scheduled to be on duty on the allocated observation days and who would be expected to participate in the interprofessional psychiatric consultations, were invited to participate, and gave their informed consent.

Patients being hospitalized for treatment in Ward A or B at the time of the observation sessions were contacted by the nursing manager or a nurse from the ward. They were informed about this study and gave their oral and written consent if they agreed to being observed during the interprofessional consultation. Some patients had to be excluded because of the following reasons: patients did not give their consent; patients were agitated and therefore not able to attend the interprofessional patient consultation; patients had a potential risk of aggressive behavior towards unknown persons, such as the observer.

### 2.2. Data Collection

The observer was present on the wards for 4 h on each observation day and took part in the interprofessional psychiatric consultations as a participating observer [35]. Furthermore, the observer was familiar with the process of interprofessional psychiatric consultation and with the events on an acute psychiatric ward. Based on this assumption and a preliminary literature review, a guide was developed for the observation sequences and simultaneously used to create the protocol [36]. The observation guide was reviewed by a team of experts from the participating institution and adjustments were made based on their expertise. Observations were recorded on the protocol by selection with a cross or keyword or free text. An overview of the content of the observation guide is provided in the table below (Table 1):

During the observation session, informal conversations before or after the interprofessional psychiatric consultations with either health care professionals or patients were recorded in the form of unstructured field notes. Furthermore, the Observing Patient Involvement in Decision-Making (OPTION) instrument was used as an additional structured data collection tool, aiming to observe the health professionals’ skills in involving patients in decisions [37,38]. The OPTION instrument was developed to assess the discursive content of a consultation and therefore focuses on an “index problem”. The evaluation process always follows the same set of rules, based on the observations made during a consultation. For guidance, the authors of the OPTION instrument provided a manual, which offers detailed description supporting the interpretation of each item-score [38].

Overall, OPTION is a frequently used observer instrument and focuses on the communication skills of health care professionals regarding decision making [37,39]. The validated instrument includes 12 items, which can be rated by the observer on a 5-point Likert scale (from 0 = not observed to 4 = the behavior shows a very high standard), shows satisfactory psychometric qualities and has been translated into various languages, including German. The individual items are designed to determine whether the health care professional clearly draws attention to a particular problem (index problem), whether the practitioner expresses that there are several solutions to the problem and explains the pros and cons of each, or what type of information the patient needs to decide.

In this study, the OPTION instrument was sequentially applied by the observer during the individual consultations according to the following criteria: a) an index problem could be identified during the individual consultation, which referred to a problem stated by either the patient or the health care professional and with clear expression that a decision needed to be made, or b) it could be recognized by the observer that the patient was involved in a decision regarding treatment by the health care professional (e.g., we have to adapt your medication and there are two possible solutions…) [37].

### 2.3. Data Analysis

In the sense of data triangulation, data were collected on two different wards with a comparable group of participants but a maximum variation of ward-related aspects, such as context and culture. The systematically collected data were in a first step analyzed individually per consultation and ward and then compared with the results of the other ward [29]. For data analysis, the triangulation method was applied using qualitative and quantitative methods. Qualitative data (e.g., written text material from the structured protocol and the unstructured field notes) were analyzed using a sequential and iterative approach. As in the first step, data were thematically analyzed, based on the topics of the observation guide and theoretical perceptions regarding communication in groups, according to Remmerswaal [40] and Merkens [41]. In a second step, additional themes were included, emerging inductively from the data. The software ATLAS.ti Version 8 (ATLAS.ti Scientific Software Development GmbH, Berlin, Germany) was used for the organization of the qualitative data.

Numeric data from the structured observation protocols (for example, time and number of health care professionals per group) and from the Observer OPTION12 scale were descriptively analyzed with frequencies using IBM SPSS Statistics Version 26 (IBM, New York, NY, United States). For interpreting the results of the Observer OPTION12 scale, the Observer OPTION12 Manual was consulted [38].

Data analysis was performed by the observer (first author) and joint meetings were held with the co-authors throughout all stages to discuss and contrast the identified codes and finally allocated to common categories.

### 2.4. Ethical Considerations

All participants (patients and health care professionals) were informed about this study in advance and gave their verbal and written consent. Clarification of jurisdiction has been obtained from the responsible ethics committee, which decided that this study does not fall under the Swiss Federal Act on research involving human beings (Request number: 2019-00175).

## 3. Results

The ethnographic study took place between December 2018 and March 2019. Overall, a total of seven (*n* = 7) four-hour period observation sessions were conducted in two acute inpatient psychiatric wards (A and B), including preparation, procedure and debriefing of regular interprofessional psychiatric consultations on a weekly basis. The sample characteristics per observation session and ward are shown in Table 2. The results of data analysis have been allocated to the following categories and subcategories: (a) architecture, including the subcategories context, preparation and form; (b) communication and interaction, with the subcategories medical talk, communication style and health care professionals’ roles; and (c) decision making, with the subcategories decision-making skills, initiative, topic, options, support and patient’s perspective.

### 3.1. Sample Characteristics

In Ward A, within 3 (*n* = 3) observation sessions, a total of 37 (*n* = 37) interprofessional psychiatric consultations were observed. In each of the 3 observation sessions, between 16 and 19 patients were consulted. Concerning the interprofessional care team, one psychiatrist with a consultant position and, additionally, two to four junior physicians within different stages of their specialization to become a psychiatrist were involved (Table 2). Furthermore, two registered nurses were mainly present, and one of them was the ward manager. A psychologist also joined the interprofessional patient consultations on an irregular basis—for example, when he was involved in the patients’ treatment. Only in one interprofessional patient consultation a relative was present.

The interprofessional patient consultations in Ward B took place twice a week, with either half of the inpatients present. Therefore, within 4 (*n* = 4) observation sessions, a total number of 34 (*n* = 34) interprofessional patient consultations were monitored. During each observation session, 10 to 13 patients and 3 to 4 junior physicians within different stages of their specialization to become a psychiatrist, participated (Table 2). Each of them was responsible for the treatment of approximately four to six patients who were hospitalized on the ward. A psychiatrist with a consultant position only joined in occasionally, when he was available. In addition, the interprofessional care team in Ward B was represented by two registered nurses, one of whom was also the ward manager, as well as by representatives of the professional groups of social workers, psychologists and/or occupational therapists, depending on their working schedules.

Overall, of the 37 patients being observed during the interprofessional psychiatric consultations in Ward A, 23% were male and 77% female and they had a mean age of 52 years (range 19–79). The most common psychiatric diagnoses were depressive disorders (51%) and substance disorders (17%). Less common diagnoses were bipolar disorders, schizophrenia, Alzheimer’s disease and anorexia. Concerning the cognitive status of the patients, qualitative descriptive data from the patient record were categorized by the authors for each patient within 3 levels: Level 1 refers to a “normal” cognitive status, which is described as “awake, conscious and oriented to all qualities and with appropriate concentration and comprehension”; Level 2 refers to a minimal impaired cognitive status, which was described in the patient record as “awake, conscious and oriented but concentration and comprehension slightly reduced”; Level 3 referred to the following description: “oriented but concentration and comprehension seriously reduced, mentally strongly constricted”. A total of 50% of the observed patients in Ward A were categorized as within Level 1, 43% within Level 2 and 7% within Level 3.

In Ward B, 64% of the 34 patients being observed during the interprofessional psychiatric consultations were male and 36% female, and they had a mean age of 41 years (range: 22–59). The most common psychiatric diagnoses were schizophrenia (36%), depressive disorders (33%) and substance disorders (18%). Other, less common disorders were stress syndrome, behavioral disorder and Alzheimer’s disease. Concerning the cognitive status, 41% of the observed patients were categorized as within Level 1, 55% within Level 2 and 4% within Level 3.

### 3.2. Architecture

#### 3.2.1. Context

The interprofessional psychiatric consultations in both wards took place in the morning approximately between 9 and 11 o’clock and staff members from the interprofessional care team were either present in their own offices or in the main ward office before the consultations. Furthermore, the main entrance door on both wards was locked during all observation sessions because some of the patients were hospitalized against their will and had to ask for permission to leave the ward.

On the one hand, the basic attitude towards the interprofessional psychiatric consultations in Ward A was according to the statements of members of the interprofessional care team especially for the psychiatrist/consultant to gain an impression of the patient’s current constitution as well as to be able to confront the patient with certain topics regarding treatment or therapy. Furthermore, nurses and junior physicians reported that they should thus receive direct information on behalf of the patient and furthermore learn from the psychiatrist. In Ward B on the other hand, the ward manager stated clearly that the interprofessional psychiatric consultation was not meant as a “learning field” but should provide a potential option for exchange between the different groups of health care professionals and the patient.

#### 3.2.2. Preparation

For preparation of the interprofessional psychiatric consultations in Ward A, topics such as adjustments to medication or the planning of future examinations were discussed in advance by the group of medical doctors (psychiatrist/consultant and junior physicians). In the meantime, nurses were updating the patient records. The patients either waited in their room for their turn or were sitting in the shared dining room and chatting with one and other.

In Ward B, for the preparation of the psychiatric consultations, interprofessional discussion also took place in the main ward office, including nurses, junior physicians, psychologists and social workers. Additionally, the nurses provided a summary report of the current patient situations and a schedule for the psychiatric consultations, which was available for the members of the interprofessional care team as well as for the patients in front of the main ward office.

#### 3.2.3. Form

**Figure 1:**
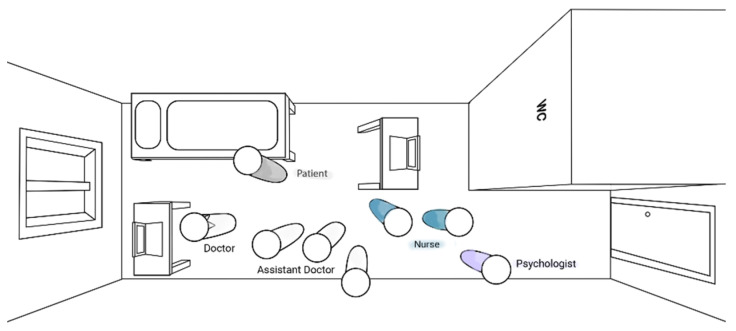
Interprofessional psychiatric consultation in the patient’s room (Ward A).

The interprofessional psychiatric consultations in Ward A had an average duration of approximately 6 min (mean) per patient and varied from 1 to 22 min. In general, they took place in the patient’s room (Figure 1). Most of the patients were accommodated in a single or a double room; and if they were visited for the consultation by the interprofessional care team in a double room, the other patient had to leave. During the consultations in Ward A, the members of the interprofessional care team stood around the patient while most of the patients were sitting on their bed or on a chair. Therefore, the psychiatrist/consultant leading the conversation and the group of junior physicians stood nearest to the patient. The other team members, such as the nurses and the psychologist, positioned themselves in the back of the room near the door.

**Figure 2:**
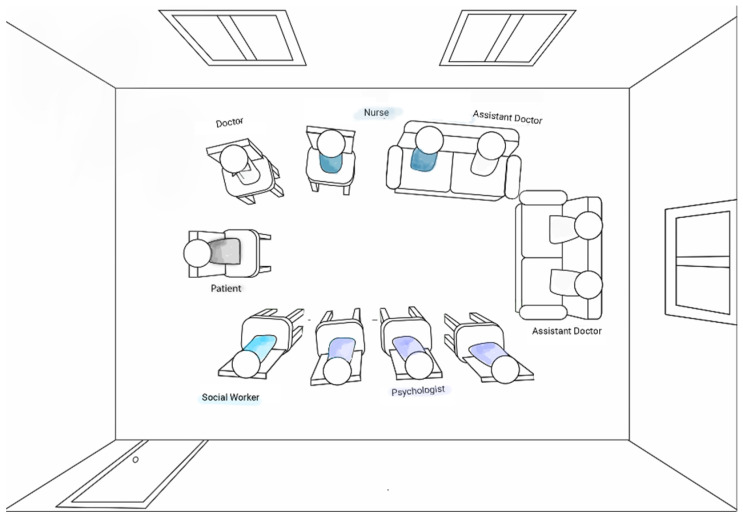
Interprofessional psychiatric consultation in meeting room with patient (Ward B).

The interprofessional psychiatric consultations in Ward B had an average duration of 8.4 min (mean) and varied from 2 to 16 min (range). For the consultations, the patients were met in a separate meeting room by the interprofessional care team. All chairs were arranged in advance as a circle and one chair was reserved for the patient (Figure 2). At the beginning of the consultations, all members of the interprofessional care team took their seats and then the patients followed one after another, as scheduled.

### 3.3. Communication and Interaction

In both wards, the conversation with the patient was started and ended by a medical doctor; in Ward A by the psychiatrist/consultant and in Ward B by either a junior physician or a psychiatrist/consultant. The psychiatrist/consultant leading the conversations in Ward A started with questions such as “how are you doing?”, “what is your current condition?”, “can you please describe your mood today?” or “what led to the crisis or the deployment?”. In Ward B instead, the conversation with the patient started with a short presentation of each member of the interprofessional care team to the patient and then the junior physician started to ask the patient about his or her current condition.

During the psychiatric consultations in both wards, the main topics discussed with the patients related to medication, and/or symptoms or referred additionally to the patient’s social, financial and/or working situation.

#### 3.3.1. Medical Talk

In relation to medication, patients on both wards were motivated to describe side effects of their current medication or they themselves asked proactively for additional medication, for example to sleep or to be able to control their body weight. The reaction of the psychiatrist/consultant or the junior physician leading the conversation was typically expressed by either agreement or disagreement, while a recognizable pattern for the observer was largely absent. Examples of some of the answers were: “Yes, we can prescribe that for you…” or “…no, you will not get that from us”. In addition, some of the patients reported side effects from medication, such as “feeling tired”, or “having back pain”, “trembling” or feeling “insecure with walking”. In such situations, these patients also received rather unspecific answers such as “this will go away within a few days” or they were advised “to do more sports” or “to lose weight”.

#### 3.3.2. Communication Style

In Ward A, observations also revealed that the conversations had a rather unidirectional character. Even when the patients were encouraged to express their feelings by the psychiatrist, he did not further elaborate on the patients’ concerns. This is illustrated by the following patient statements and reactions of the psychiatrist/consultant, as one patient stated: “…I am feeling lonely” or another who mentioned “…I am worrying about my condition and not being able to get better…”. The psychiatrist/consultant answered: “…there are many nice people here, you are certainly not lonely…” or “…you do not have to worry, you are in good hands here…”. Additionally, the other health care professionals (mainly the junior physicians and the nurses) present barely interacted with the patient and had no recognizable task during the consultations, besides taking notes for updating the patient record.

In Ward B, the junior physicians were predominantly aware of their language and tried to adapt diagnosis-related expressions into a more comprehensible language, other than medical terminology. For example, instead of asking the patient if she/he is having “depressive symptoms”, they used the expression of “feeling unhappy”. Regarding medication, commercial drug names were also used without further information on their effects and side effects. However, the patients did not ask, which either indicates that they already knew the names or did not bother to ask. Overall, in Ward B, the patient was addressed by different persons present and the conversations were more dynamic, meaning that psychologists or social workers also had the opportunity to discuss their concerns with the patient and vice versa.

#### 3.3.3. Health Care Professionals’ Roles

The health care professionals’ roles as silent listeners or as “keepers of the minutes” in Ward A, except for the psychiatrist/consultant, were justified by the members of the interprofessional care team by the statement that they all would have the same level of knowledge about the patient’s situation. Furthermore, the psychiatrist/consultant referred to the interprofessional care team as “his entourage”. On the contrary, patients reported that it felt very uncomfortable, especially at the beginning of a hospitalization, “…to be confronted with such a large group of people in one’s own room and to be unable to identify anyone apart from the psychiatrist”. Furthermore, patients mentioned: “Staff members do not introduce themselves…” and “…it is not possible to recognize who belongs to which professional group, because so many people are present in the room”.

In Ward B, social workers, occupational therapists and/or psychologists were substantially involved in the conversations with the patient when specific topics regarding the living and/or working situation of the patient was at stake. This was the case whether a new housing solution had to be found, the patient was allowed to go home on a trial basis for a few days or outpatient solutions had to be chosen after hospitalization. If requested by the patient, they also provided information on planned measures, such as schedules for therapies, additional treatment such as sports or planning a daily structure during the conversations. Overall, an interprofessional approach was noticeable through observation and it became evident that the patients gained supplementary information from these contributions on non-medical aspects. This became obvious during the observation, for example, when a patient described “…that he felt bored”. The occupational therapist was then able to present the occupational services and to clarify with the patient, what would be of interest to him. In another situation, the social worker offered the possibility that the upcoming change of residence could also be organized from the clinic. This contributed to considerable relief on the patients’ behalf.

### 3.4. Decision Making

In both wards, in less than half of the interprofessional psychiatric consultations, an “index problem” could be notified which required a decision—and, in those cases, the OPTION instrument was completed. The defining features of a decision-making situation according to Elwyn et al. [38] include a specific problem which needs to be solved, a choice between several valuable options for a treatment which are presented to the patient, or the patient is expected to participate in a decision about the management of his/her current condition and where possible solutions are considered more in detail. While in Ward A, approximately 41% (*n* = 15) of the observed psychiatric consultations were related to a decision, this was the case in 35% (*n* = 12) in Ward B.

#### 3.4.1. Decision-Making Skills

A total score was calculated for the results of the OPTIONscale by adding the scores of each item and then standardizing the sum to a value between 0 and 100. A high score indicates that the communication competencies of the health care professional observed referred to “a good standard” regarding SDM [37]. Hence, the average score of the two wards ranked among the lower ranges of total scores and showed comparable values. The mean of the total score in Ward A was 24 (range 8–42) and in Ward B 25 (range 6–47), which indicates according to the OPTION manual [38] a “minimal attempt of patient involvement”. The individual score of each of the 12 items per consultation with a decision, varied from “not observed” (score = 0) to “observed with a high standard” (score = 4).

#### 3.4.2. Initiative

In Ward A, in 8 out of 15 interprofessional psychiatric consultations, the psychiatrist/consultant directed attention to a specific problem requiring a decision. A total of 2 out of the 15 patients where a decision had to be made were prepared with notes for the consultation and accordingly guided the discussion with the psychiatrist/consultant by presenting their requests. In the remaining five psychiatric consultations with a decision-making situation, the initiative to take a decision emerged implicitly, during the course of the consultation.

In Ward B, in 10 out of 12 decision-making situations, the junior physician or the psychiatrist/consultant leading the conversation drew attention to a problem that required a decision.

#### 3.4.3. Topic

Most of the decision-making situations in Ward A involved medication. Most frequently, the attending psychiatrist/consultant and the junior physician discussed the adjustment of a medication and asked the patient about side effects or irregularities. Sometimes it was not the patient who answered, but one of the junior physicians instead. In other situations, a decision was made by the psychiatrist/consultant to adjust the dosage or to change the medication, but the patient was not involved further in the process. In two of the consultations, the patients themselves made suggestions regarding modification of medication, for example: “I would like to try medication XY instead…” or “In my experience, the dosage of XY micrograms was last time considered as the optimum”. Since there was no objection to the patient’s suggestions from a medical point of view either, the patient’s wishes were taken into account and the medication was adjusted accordingly.

In Ward B, medication was the main topic in six consultations, while in the other four consultations, additional therapy (e.g., psychosocial education) or the decision to leave the ward for a few hours was discussed with the patient.

#### 3.4.4. Options

In one-third (*n* = 5) of the decision-making situations during a consultation in Ward A, the psychiatrist/consultant leading the conversation stated clearly that there were options regarding problem solving, and pros and cons had to be discussed in further detail. This was the case, for example, as the psychiatrist suggested additional therapy to medication, such as electroconvulsive therapy (EKT). Thus, the patient worried about the side effects and asked about the pros and cons regarding the EKT therapy. As a consequence, the psychiatrists/consultant not only provided the requested information verbally but offered the opportunity to discuss this further in a follow-up consultation.

In Ward B, options regarding problem solving as well as pros and cons were discussed with the patients in more than a half (*n* = 7) of the 12 consultations with a decision to make. In one exemplary situation, the physician assistant offered two different types of medication to be used as needed by the patient, with the same effect. The patient was therefore encouraged to choose his/her preferred type of medication and to adjust the dosage according to his/her needs, by requesting the medication when needed.

#### 3.4.5. Support

Furthermore, in 14 of 15 decision-making situations in Ward A, the psychiatrist/consultant leading the conversation did not assess the patients’ preferred approach for receiving information—for example, with printed material. Information was provided barely verbally. Regarding the presentation of the options to solve the problem addressed and to mention the possibility to take no action, this behavior was only observed in 4 out of 15 decision-making situations.

In Ward B, 3 out of 12 patients were directly asked about their preferences for receiving additional information material, such as printed information about therapeutic services or medication schedules. Options to solve the problem were presented to 7 out of 12 patients and these patients also received an explanation regarding the pros and cons of the options by the junior physician leading the conversation.

#### 3.4.6. Patient’s Perspective

The psychiatrist/consultant in Ward A explored the patient’s perspective and concerns regarding a decision in less than half (*n* = 6) of 15 decision-making situations. With regard to the majority of the decision-making situations, the psychiatrist/consultant did not explore the patient’s understanding of the information provided, he did not offer the patient the opportunity to ask questions and did not explore the patient’s preferred level of involvement in decision making.

In 8 out of 12 decision-making situations in Ward B, the patients were asked about their expectations regarding the management of the problem and subsequently their fears were also elaborated by the health care professional leading the conversation. Two patients were actively asked about their understanding of the provided information about the possible options. The opportunity to ask questions was offered to 1 of the 12 patients and none of them was asked about his/her preferred level of involvement in decision making.

## 4. Discussion

The results of our study show that the interprofessional psychiatric consultations on the two wards observed differed significantly in terms of the place and form in which they were carried out, as well as the composition of the professional groups represented and their roles during the consultation. However, on the contrary, the extent to which patients were actively involved in the discussion as well as in decision making was comparable on both wards and depended on individual factors, such as the health care professionals’ communication style as well as the patients’ personal initiative to be engaged.

The contextual and cultural factors of the interprofessional psychiatric consultations differed substantially between the two participating wards, not only in terms of the structural form and location they took place, but also regarding the composition and the roles of the interprofessional care team involved. These differences could be explained due to the different ward-related concepts, which should be carefully reflected upon with regard to the ward-specific application and interpretation of an interprofessional team approach [26,42]. While the interprofessional collaboration in Ward A seemed rather programmatic and could be described as “the presence of different professional groups with no responsibility and with medical leadership”, the interprofessional approach in Ward B could be specified as “the partial involvement of several professional groups with medical leadership” [26]. Hence, a benefit for the patients being confronted with a rather large group of health care professionals without any specific task during the consultation, especially in Ward A, could not be identified by observation. However, when it comes to decision making in psychiatric care, the necessity of interprofessional collaboration, the involvement of multiple stakeholders as well as cultural adaptions are described as being helpful for the patients and should therefore be promoted [8,10,23,43].

We also found the content of the conversations in both wards to be predominantly medically oriented, by focusing on the patient’s condition, medication or symptoms. These observations could be explained by the fact that on both wards, the consultations were directed by the group of medical doctors (e.g., psychiatrists or physician assistants). The other professional groups present had a more subsidiary role during the consultations. While on Ward B the therapeutic health care professionals (social workers, psychologists and occupational therapists) added to the conversations, when topics such as additional treatment or housing were at stake, the nurses on both wards mainly acted as “silent listeners” and “keepers of the notes”. Other studies point out that the roles of the health care professionals may vary across the patient’s illness trajectory or setting [10]. The reason why nurses, in particular, barely contributed could be due to the fact that this professional group has not yet found a partnership position in the interprofessional collaboration within the Swiss psychiatric inpatient setting. In the future, however, this would be an essential prerequisite for psychiatric nurses to be able to increasingly advocate for the interests of patients and provide them with advice [10,44].

Furthermore, we observed that on both wards, the extent to which patients were actively engaged in decision making was low according to the average score of the OPTION instrument (Ward A = 24; Ward B = 25) and not consistent. The latter became evident because the range of the total score varied considerably, between 8 and 42 in Ward A, and between 6 and 47 in Ward B. These observations are comparable to the results of a review by Couët et al. [39], which reported patients not being involved with consistency during medical consultations in different clinical contexts. One possible explanation regarding the distribution of the overall score in both wards in our study could be the fact that the medical doctors leading the consultations did not follow a structured approach to facilitate patient involvement in decision making [10,39].

Another explanation regarding the inconsistency in patient involvement could be the patient’s personal initiative to be engaged during the consultation. While a few patients actively participated by making clear demands or asking specific questions, most others were more passive and merely responded to the health care professionals’ questions. While previous work has found psychiatric patients wanting to be explicitly involved in care and decision making [8,45], others argue the patients’ preferences towards involvement may vary according to their stage of illness and their experiences [10]. The extent to which patients engage in decision making depends, among other things, on the relationship with the health care professional and is described as a reciprocal interaction [6]. This interaction, however, may also be influenced by the pathophysiology of cognitive and behavioral brain functions because of the psychiatric disorders present. In this respect, we can refer to the contribution that prefrontal brain regions have in the assessment of rewarding, which underlies subsequent decision making and goal-directed behavior [6,46].

The patients’ decision-making capacity (e.g., decision-making ability) is reported frequently as a barrier to SDM regardless of the fact that patients have a legal right to be informed about the effects and side effects of therapy or treatment and evidence shows current management strategies (e.g., pharmacotherapy and psychotherapy) as only partially efficient [47,48,49,50]. Hence, according to Couët et al. [39], patients themselves are part of the solution for better involvement and should be supported and trained in bidirectional communication together with the health care professionals. The active involvement of patients in treatment decisions can create a feeling of being valued and being recognized as an individual rather than a diagnosis [49,51].

### Strengths and Limitations

Addressing strength and limitations, the ethnographic approach of this study provided a situational perspective into the daily practice of interprofessional psychiatric consultations in an acute psychiatric inpatient setting. By applying participant observation as a research strategy, both the perspectives of patients and health care professionals were included, and the natural course of the consultations was only minimally impaired by this method. Concurrently, the ethnographic approach could also have biased the actions of both the health care professionals and the patients, as they might have been on their best behavior, for example by using a more patient-focused language or by being more compliant. However, the results of this study can help to understand the nature of clinical situations, where communication between health care professionals and patients takes place and where quality improvement could be tailored. Further research is needed to assess the patients as well as the health care professionals’ personal attitudes on SDM and PCC and their impact on organizational culture and daily practice in the psychiatric inpatient setting.

## 5. Conclusions

Despite the fact that the legal and ethical imperative of SDM and PCC in health care are widely accepted, the results of our study in the psychiatric inpatient setting show that the patient perspective still has a subordinate relevance to the perspective of the health care professionals and is not yet implemented as a quality indicator regarding the provision of care. Further efforts are required that promote a significant transformation of the organizational culture, which should not only have an impact on the persons involved (e.g., health care professionals and patients), but also on the ward and institutional level. In particular, those responsible on the wards should increase their efforts to ensure that ward concepts also contain clear information on how attitudes and culture could be reflected in the sense of SDM and PCC. Ward culture and health care professionals’ attitudes should become apparent in the interactions between patients and health care professionals around treatment and care. Furthermore, patients’ concerns and preferences must become more visible, and it would therefore be necessary to ask the patients how interprofessional psychiatric consultations work for them and what they would expect from a participatory collaboration with the health care professionals present. Further research should also focus on different health care professionals in addition to medical doctors. The aim would be to clarify the possible contribution of the individual groups of health care professionals about the needs identified by the patients. For further improvement, rather than focusing on contextual factors, communication training for all members of the interprofessional care team as well as for the patients is mandatory. Regarding communication training to promote patient involvement in the psychiatric inpatient setting, patients should be empowered to communicate bidirectionally with the health care professionals: either by clarifying their preferred level of involvement, by receiving opportunities to prepare for the consultations or by being encouraged to ask questions.

## Figures and Tables

**Table 1:** Observation guide: Aspects being observed during interprofessional psychiatric consultations.

Aspects Being Observed	Specification
Contextual and cultural aspects	Specification of location where the interprofessional patient consultation took place
	The duration of the interaction with each patient during the interprofessional patient consultation
	Roles of the individual health care professionals present
	Visualization of the room situation and position of the patient and the different health care professionals present
Communication and interaction	Verbal description of the main problem by the patient and/or health care professional
	Specific language (medical or adapted to the patient’s needs) used by the health care professionals
	Speaking time of the patient and the different health professionals present
	Extent of participation in the conversation by the persons present, e.g., who was leading the conversation
	Documentation of agreements or decisions regarding therapy or treatment by a health care professional
	Discussions between the health professionals present with/without involving the patient
Decision-making skills	The Observing Patient Involvement in Decision-Making (OPTION) instrument summarizing the skills of health professionals in the decision-making process in a structured way, if a treatment decision had to be made

**Table 2:** Number of participants per observation session.

Setting	Group of Participants	Range
Ward A	Patients	16–19
	Junior physicians	3–5
	Nurses	1–3
	Psychologist/Consultant	0–1
Ward B	Patients	10–13
	Junior physicians	3–4
	Nurses	2
	Psychologists/social workers/occupational therapists	2–5

## Data Availability

The data presented in this study are available on request from the corresponding author due to privacy restrictions of the participating hospital.
